# Establishment and Molecular Characterization of a Human Stem Cell Line from a Primary Cell Culture Obtained from an Ectopic Calcified Lesion of a Tumoral Calcinosis Patient Carrying a Novel *GALNT3* Mutation

**DOI:** 10.3390/genes16030263

**Published:** 2025-02-24

**Authors:** Simone Donati, Gaia Palmini, Cinzia Aurilia, Irene Falsetti, Francesca Marini, Gianna Galli, Roberto Zonefrati, Teresa Iantomasi, Lorenzo Margheriti, Alessandro Franchi, Giovanni Beltrami, Laura Masi, Arcangelo Moro, Maria Luisa Brandi

**Affiliations:** 1 Department of Experimental and Clinical Biomedical Sciences, University of Florence, Viale Pieraccini 6, 50139 Florence, Italy; simone.donati@unifi.it (S.D.); cinzia.aurilia@unifi.it (C.A.); irene.falsetti@unifi.it (I.F.); gianna.galli@unifi.it (G.G.); teresa.iantomasi@unifi.it (T.I.); 2 FirmoLab, Fondazione F.I.R.M.O. Onlus and Stabilimento Chimico Farmaceutico Militare (SCFM), 50141 Florence, Italy; gaia@fondazionefirmo.com (G.P.); francesca.marini@fondazionefirmo.com (F.M.); zonefrati@gmail.com (R.Z.); 3 Stabilimento Chimico Farmaceutico Militare (SCFM)—Agenzia Industrie Difesa (AID), 50141 Florence, Italy; lorenzo.margheriti@aid.difesa.it (L.M.); arcangelo.moro@aid.difesa.it (A.M.); 4 Department of Translational Research and of New Technologies in Medicine and Surgery, University of Pisa, 56126 Pisa, Italy; alessandro.franchi@unipi.it; 5 Department of Orthopaedic Oncology and Reconstructive Surgery, Azienda Ospedaliero, Universitaria Careggi, 50134 Firenze, Italy; giovanni.beltrami@meyer.it; 6 Metabolic Bone Diseases Unit, University Hospital of Florence, AOU Careggi, 50139 Florence, Italy; masilau@aou-careggi.toscana.it

**Keywords:** tumoral calcinosis, ectopic calcification, stem cells, mesenchymal stem cells, embryonic stem cells, sphere formation assay, three-dimensional cell culture, microRNAs, epigenetics

## Abstract

Background/Objectives: Tumoral calcinosis (TC) is an extremely rare inherited disease characterized by multilobulated, dense ectopic calcified masses, usually in the periarticular soft tissue regions. In a previous study, we isolated a primary cell line from an ectopic lesion of a TC patient carrying a previously undescribed *GALNT3* mutation. Here, we researched whether a stem cell (SC) subpopulation, which may play a critical role in TC progression, could be present within these lesions. Methods: A putative SC subpopulation was initially isolated by the sphere assay (marked as TC1-SC line) and characterized for its stem-like phenotype through several cellular and molecular assays, including colony forming unit assay, immunofluorescence staining for mesenchymal SC (MSC) markers, gene expression analyses for embryonic SC (ESC) marker genes, and multidifferentiation capacity. In addition, a preliminary expression pattern of osteogenesis-related pathways miRNAs and genes were assessed in the TC1-SC by quantitative Real-Time PCR (qPCR). Results: These cells were capable of differentiating into both the adipogenic and the osteogenic lineages. Moreover, they showed the presence of the MSC and ESC markers, confirmed respectively by using immunofluorescence and qualitative reverse transcriptase PCR (RT-PCR), and a good rate of clonogenic capacity. Finally, qPCR data revealed a signature of miRNAs (i.e., miR-21, miR-23a-3p, miR-26a, miR-27a-3p, miR-27b-3p, and miR-29b-3p) and osteogenic marker genes (i.e., *ALP*, *RUNX2*, *COLIA1*, *OPG*, *OCN*, and *CCN2*) characteristic for the established TC1-SC line. Conclusions: The establishment of this in vitro cell model system could advance the understanding of mechanisms underlying TC pathogenesis, thereby paving the way for the discovery of new diagnostic and novel gene-targeted therapeutic approaches for TC.

## 1. Introduction

Tumoral calcinosis (TC) is an extremely rare congenital disease characterized by lobular, densely calcified, ectopic masses, usually developed in childhood, and typically forming in the periarticular spaces of soft tissues (i.e., hips, elbows, and shoulders) [1]. Initially, these calcified masses grow slowly and are asymptomatic, but progressively they could reach large sizes and drastically alter joint function and body movement. Dental abnormalities, such as obliteration of the pulp cavity and pulp calcifications, are often associated with this disease; testicular microlithiasis, neuronal calcifications, and ocular manifestations, including calcifications in the cornea, retina, and eyelid, have been reported as part of the disease as well [2,3,4,5]. Main biochemical abnormalities are persistent hyperphosphatemia secondary to increased renal proximal tubule Pi reabsorption and hypervitaminosis D. This can lead to pathological development of large periarticular calcified masses in the soft tissues, weighing up to 1 kg [6,7]. At surgery, TC lesions are typically cystic in appearance and contain a chalky material known as “calcium hydroxyapatite (HA) crystals”, along with calcium phosphate and amorphous calcium carbonate [8].

TC has been primarily reported in individuals originating from Africa and the Middle East [6,9]. No published study has reported the overall prevalence of TC. Considering the paucity of genetically documented cases worldwide, TC is classified as extremely rare. Patients are often incidentally uncovered while undergoing routine imaging investigations that are not related to TC.

Despite TC initially being described as having an autosomal dominant inheritance pattern, the recent identification of the genetic defects underlying all forms of FTC, it was established as an autosomal recessive disorder [10,11,12,13].

At germline level, TC is characterized by loss-of-function genetic alterations in genes coding for phosphatonins or proteins involved in their activity control, with three major mutated genes: the *fibroblast growth factor 23* (*FGF23*) [12,14,15,16], the *UDP-N-acetyl-a-D-galactosamine*: *polypeptide N-acetyl-galactosaminyltransferase 3* (*GALNT3*) [3,4,11,13,17,18], and *Klotho*, a co-receptor for the end-organ bioactivity of FGF23 [17].

Regarding the *GALNT3* gene, mapping analysis on patients showed that TC was associated with homozygote loss-of-function mutations of this gene, which encodes a ubiquitously expressed glycosyltransferase responsible for the initial O-glycosylation of FGF23 at specific serine and threonine residues [11,19,20,21]. Most congenital glycosylation-related disorders are caused by abnormal N-glycosylation and are characterized by a wide range of clinical manifestations [22]. However, TC is the only one that results from impaired O-glycosylation and appears to be associated with ectopic calcifications. In particular, as a consequence of *GALNT3* inactivating mutations, proteolytic protection of FGF23 by O-glycosylation wanes, thereby decreasing the phosphaturic activity of FGF23.

To date, more than 10 different mutations in *GALNT3* have been reported, all of them predicted or found to cause loss of GALNT3 function [3,4,11,13,23,24,25,26,27]. Alteration in this enzyme has not only been associated with TC but also with another autosomal recessive syndrome, the hyperostosis–hyperphosphatemia syndrome (HHS), also characterized by increased levels of serum phosphate [28,29,30]. Despite HHS and TC patients carrying the same mutated gene, these disorders manifest phenotypically in different tissues, bone, and skin, respectively.

MicroRNAs (miRNAs) are short non-coding RNAs (~19–25 nucleotides in length) that are able to negatively control the expression of several target genes at the post-transcriptional level, exerting a pivotal regulatory role in numerous biological processes [31]. In 2002, studies performed by Calin and colleagues demonstrated for the first time that a signature of abnormal miRNA expression was responsible for the development of primary chronic lymphocytic leukemia [32]. Since then, several studies have described that miRNAs’ dysregulation is involved in the tumorigenesis of several tumors [33,34]. Over the last few years, various studies have identified that miRNAs are also important modulators of bone development and growth, and an aberrant expression of specific miRNAs has been tightly associated with onset and progression of various skeletal disorders, such as osteoporosis, osteonecrosis, osteosarcoma, and bone tumor metastasis [35]. Therefore, identifying a disease-associated distinguishing miRNA signature could represent a promising theragnostic target for bone disorders for overcoming some challenges about the dose-related adverse effects and the delivery modalities in conventional treatments [36,37].

In a healthy organism, the presence of a subset of cells with stemness features, such as self-renewal and the capacity to differentiate toward multiple cell lineages, is important to maintain normal tissue homeostasis [38]. The development, maintenance, and repair of bone tissue are supported by stem properties of resident stem cells (SCs) [39]. Likewise, within the tumor bulk has been described the presence of a cancer SCs (CSCs) subset that exhibits characteristics similar to normal SCs and is responsible for the initiation and maintenance of the disease. Moreover, CSCs have the intrinsic ability to resist standard intervention, which generally consists of chemo- and radiotherapy due to enhanced expression of drug-resistance ATP-binding cassette (ABC) transporters, deregulation of several signaling transduction pathways involved in cell survival and proliferation, enhanced epithelial to mesenchymal transition (EMT), and increased immune escape and DNA repair mechanisms [40,41,42,43].

In this light, research on SCs holds great potential that could lead to a paradigm shift toward the treatment of bone-related diseases and the understanding of the underlying pathogenetic mechanisms.

In 1992, pioneering in vitro studies by Reynolds et al. developed a protocol to isolate multipotent cells with suspected stem-like features from the striatum of adult mouse brain [44,45]. The protocol was based on the ability of SCs to form spherical colonies when grown under nonadherent conditions. Similar methods were employed by Gibbs and colleagues to study a stem-like cell subpopulation in bone sarcomas [46]. Previous studies by our Research Group adapted this technique to isolate and characterize osteosarcoma (OSA)-CSC cell lines derived from finite primary cell cultures of different conventional OSA types [47].

Here, we applied the abovementioned method to establish and characterize, for the first time, an SC subpopulation line from a primary cell culture obtained directly from an ectopic calcified lesion of a TC patient carrying an undescribed *GALNT3* gene variation. We also explored the epigenetics basis associated with TC progression, aiming to assess the expression pattern of selected miRNAs and genes related to osteogenic differentiation.

## 2. Materials and Methods

### 2.1. Isolation of a Primary Cells Culture of TC

In a previous study by our Research Group [48], a TC primary cell culture was obtained from a biopsy of subcutaneous adipose tissue from an ectopic calcified lesion, collected at the “Unità Ortopedia Oncologica e Ricostruttiva”, AOUC Careggi, Florence. After the collection in a sterile environment, through fine-needle aspiration, the tissue sample was immediately located in an antibiotic-supplemented media pH 7.4, and transferred to the laboratory for processing. The primary cell culture was generated after collagenase treatment in a modified Ham’s F12 Coon’s medium (Sigma-Aldrich, St. Louis, MO, USA) supplemented with 20% fetal bovine serum (FBS), 1% penicillin/streptomycin solution, and collagenase type II (Sigma-Aldrich, St. Louis, MO, USA), at 37 °C until complete digestion. After centrifuging, the cell pellet was subjected to mechanical dispersion and sedimented by centrifugation. Cells were then cultured as a monolayer in a humidified atmosphere of 5% CO_2_ in air at 37 °C, in the growth medium (GM) (i.e., Ham’s F12 Coon’s modification medium supplemented with 10% FBS). GM was refreshed every 3 days. The obtained TC primary cell culture was named as “TC1”. Once at confluence, TC1 cells were subcultured or harvested using 1× trypsin-EDTA.

### 2.2. Sphere Formation Assay

SCs were isolated, from the established primary culture, through a sphere forming–based assay, as described in a previous work by our Research Group [47]. When 90% confluence was reached in GM, monolayer cells were detached into a single-cell suspension using 1× trypsin-EDTA. Then, cells were plated at a density of 40,000 cells/well in ultra-low attachment six-well plates (Corning Inc., Corning, NY, USA) in 2× Ham’s F12 Coon’s modification medium supplemented with 2% sterile methylcellulose, putrescine (100 μM), progesterone (20 nM), sodium selenite (30 nM), transferrin (25 μg/mL), insulin (20 μg/mL), and the growth factors human recombinant epidermal growth factor (EGF, 10 ng/mL) and human basic fibroblast growth factor (b-FGF, 10 ng/mL). These latter growth factors were added every 3 days. All reagents were purchased from Merck (Burlington, MA, USA). After 14 days of culture under nonadherent conditions, spherical floating colonies composed of more than 50 cells were defined spheres, and then isolated and plated in 60 mm diameter tissue dishes in GM, under normal attachment conditions, thereby establishing the TC SCs, named as “TC1-SCs”. When 80–90% confluence was reached, TC1-SCs were harvested and then placed into a petri dish (100 mm diameter) for the successive characterization analyses.

### 2.3. Stem Cell Phenotype Characterization

The stem-like phenotype of the TC1-SCs line was evaluated by colony forming unit (CFU) assay, immunofluorescence staining, gene expression analyses, and their capacity to differentiate toward the adipogenic or the osteogenic lineages.

#### 2.3.1. Colony Forming Unit (CFU) Assay

When confluence was reached, cells were dissociated with 1× trypsin-EDTA and reseeded in fresh modified medium of Ham’s F12 Coon’s supplemented with 20% FBS, 100 IU/mL penicillin and 100 μg/mL at a seeding density of 1000 cells/cm^2^ in 100 mm diameter dishes. Cells were grown at 37 °C in humidified air with 5% CO_2_ for 4 weeks until colonies’ formation. The medium was refreshed once a week. This experiment was performed in triplicate. The colonies were stained with 1% toluidine blue and estimated via inverted microscope (LSM-900, ZEISS, Oberkochen, Germany). The total number of colonies composed of more than 50 cells was counted to evaluate the colony forming efficiency, as follows:

(Number of counted colonies/number of cells seeded) × 100.

#### 2.3.2. In Vitro Adipogenesis Differentiation of TC1-SCs

The TC1-SCs line was plated in a 24-well plate in GM and grown up to 80–90% confluence. Then, adipogenesis was induced by using a specific adipogenic medium (AM) consisting of GM supplemented with 1 μM bovine insulin, 1 μM dexamethasone, and 500 μM isobutylmethylxanthine. The AM was replaced every 2–3 days. Different stages of adipogenesis were monitored by microscopic observation. The adipogenic phenotype was evaluated for 30 days, with respect to control cells cultured in GM, by conventional staining of intracellular lipid droplets by a freshly prepared solution of Oil Red-O. Immunostained mature adipocytes containing lipid droplets were visualized under bright field microscopy (LSM-900, ZEISS, Oberkochen, Germany).

#### 2.3.3. In Vitro Osteogenic Differentiation of TC1-SCs

The TC1-SCs line was plated on a 24-well plate in GM to reach 80–90% confluence in each well. Subsequently, cells were induced to differentiate toward the osteogenic lineage by using a specific osteogenic medium (OM) consisting of GM supplemented with 10 nM dexamethasone, 10 mM β-glycerol phosphate, and 200 μM sodium L-ascorbyl-2-phosphate. OM was replaced every 2–3 days. After 35 days, differentiated cells were characterized for the osteoblastic phenotype, as follows. After washing with Dulbecco’s phosphate buffered saline (DPBS), cells were fixed in 4% paraformaldehyde (PFA)/DPBS for 10 min, and subsequently rinsed three times with ultrapure distilled H_2_O. Then, cells were stained for alkaline phosphatase (ALP) with a specific dye mixture composed by Solution A, prepared by dissolving 5 mg naphthol AS-MX phosphate sodium salt in 1 mL dimethyl sulfoxide (DMSO), and Solution B, prepared by dissolving −40 mg Fast Red Violet LB salt in 50 mL Tris-HCl Buffer 280 mM, pH 9.0, which were mixed thus forming the working Solution. One milliliter of working Solution was added to each well for 30 min at 37 °C in humidified air with 5% CO_2_. ALP-positive cells were stained in red, and nuclei were counterstained with methyl green nuclear dye. For assessing the presence of HA crystal deposits, fixed cell samples were stained with 1% silver nitrate solution and exposed to ultraviolet light for 4 h. Then, the unbound silver was removed by rinsing ultrapure distilled H_2_O (three times). ALP-positive cells and HA crystals were observed under bright field microscopy (LSM-900, ZEISS, Oberkochen, Germany).

#### 2.3.4. Immunofluorescence Staining of Mesenchymal Stem Cell Markers

Immunofluorescence staining on fixed TC1-SCs, as previously described in Section 2.3.3, was used to examine the presence of mesenchymal SC (MSC) markers, by incubating with primary antibodies to detect CD34, CD44, CD45, CD90, and CD105 expression. After fixation, membrane cells were permeabilized by incubation with a 0.2% Triton X-100 solution in DPBS for 30 min at 37 °C in humidified air with 5% CO_2_. Cells were washed with DPBS (three times) and incubated with 1:100 RNase in 2% bovine serum albumin (BSA)/DPBS for 30 min at 37 °C in humidified air with 5% CO_2_ to prevent the nonspecific antibody binding. Subsequently, cells were washed in 2% BSA/DPBS three times and incubated with specific primary antibodies Anti-CD44 (Abcam, Cambridge, UK), anti-CD105 (Invitrogen, Carlsbad, CA, USA), anti-CD90 (Abcam), anti-CD34 (Abcam), and anti-CD45 (Abcam) in a humidified chamber at 4 °C overnight. Afterward, the antibody excess was washed away, and samples were incubated with an Alexa Fluor 488-conjugated Superclonal Secondary Antibody (Invitrogen), in the dark in a humidified chamber at RT for 45 min. Lastly, cells were washed with DPBS several times and 2% BSA/DPBS and nuclear counterstain was performed through 1:100 Propidium iodide in DPBS. For negative control, cells were incubated only with the secondary antibody. Stained cells were observed with 10× and 20× at RT under a Laser Scanning Confocal Microscopy (LSM 900, ZEISS, Oberkochen, Germany).

#### 2.3.5. Expression of the Embryonic Stem Cell (ESC) Genes by Real-Time PCR

RNA of TC1-SCs and TC1 was isolated by QIAzol Lysis Reagent (Qiagen, Hilden, Germany, 79306). RNA concentration and purity were assessed spectrophotometrically by measuring 260 nm and 280 nm absorbances. The integrity of the total RNA was evaluated on a standard 1% agarose gel. The expression of ESCs and of pluripotency-related marker genes (*POU5F1*, *Nanog*, and *KLF4*), was assessed both on TC1 and TC1-SC cells, cultured in GM, through a two-step qualitative reverse transcription PCR (RT-PCR). Four hundred nanograms of RNA was initially reverse transcribed by using the QuantiTect Reverse Transcription kit (Qiagen, Hilden, Germany, 205310). *β-Actin* was used as internal control gene. Used primers (Integrated DNA Technologies, Coralville, IA, USA) are shown in Table 1.

### 2.4. Expression of the Osteogenic Marker Genes

Expression of the osteogenic phenotype was evaluated both on TC1 and TC1-SCs and compared to a primary wild-type subcutaneous SC line obtained from a normal subject (named “PA24”). In this regard, expression of known osteogenic marker genes (i.e., the early markers of osteoblastic differentiation, such as *ALP*, *runt-related transcription factor-2* (*RUNX2*), and *type I procollagen* (*COLIA1*) [49]; *osteoprotegerin* (*OPG*), a protein secreted by osteoblasts that inhibits osteoclast differentiation [49]; *osteocalcin* (*OCN*), a marker expressed in the later stages of osteogenesis [49]; and *connective tissue growth factor* (*CTGF*), also known as “*CCN family member 2* (*CCN2*)”, a master regulator of osteoblastogenesis [50]), was evaluated in all the three cell lines induced to differentiate toward the osteogenic lineage, up to 21 days from osteogenic induction. At 0, 4, 7, 14, and 21 days from osteogenic induction, cells were detached and pelleted for RNA extraction. Total RNA and cDNA were isolated and prepared as described in Section 2.3.5. Quantitative Real-Time PCR (qPCR) analyses were performed using specific primers and probes (Table 1) designed by IDT integrated DNA technologies, following the manufacturer’s instruction. *Glyceraldehyde-3-phosphate dehydrogenase* (*GAPDH*) was used as a reference gene for quantifying gene expression, according to a previous work [48]. qPCR was conducted using a GoTaq^®^ qPCR Master Mix (Promega Corporation, Wisconsin, USA) on a Rotor-Gene^®^ Q Real-Time PCR cycler (QIAGEN, Hilden, Germany) in TaqMan technology. All points for standard curves and unknown samples were analyzed in duplicate.

### 2.5. microRNA Expression Analysis

The expression profile of a selected panel of six miRNAs (miR-21, miR-23a-3p, miR-26a, miR-27a-3p, miR-27b-3p, and miR-29b-3p) in TC1, TC1-SC, and PA24 cell lines was detected using a qPCR-based approach. Briefly, total RNA highly enriched for small RNAs was extracted using the miRVana miRNA isolation Kit (Invitrogen, Waltham, MA, USA), according to the manufacturer’s instructions. cDNA for each miRNA was synthesized from the total miRNA fraction using the TaqMan MicroRNA Reverse Transcription kit (Applied Biosystem, Foster City, CA, USA) and the associated mature miRNA-specific primers from TaqMan MicroRNA Assays (Applied Biosystem, Foster City, CA, USA), according to the manufacturer’s protocol. During the amplification step, targets were amplified by using TaqMan Universal Master Mix II, with UNG (Applied Biosystem, Foster City, CA, USA). For the RT reaction, the reaction mixture was incubated at 16 °C for 30 min to anneal primers, at 42 °C for 30 min for the extension of primers on miRNA and the synthesis of the first cDNA strand, and at 85 °C for 5 min to stop the reaction. The RT reaction products were then stored at −20 °C until use. Small nucleolar RNA U6 was used as the internal control standard for data normalization. The cycle threshold (Ct) was calculated by using the fixed threshold setting. The relative expression of each analyzed target miRNA was calculated by using the 2^−∆ΔCT^ method. In detail, to calculate target miRNA expression normalized with respect to the reference small nucleolar U6, the comparative C_t_ (ΔΔC_t_) method was used [51], with fold change = 2^−[(Ct miRNA of interest−Ct small nucleolar U6) differentiated cells−(Ct miRNA of interest−Ct small nucleolar U6) undifferentiated cells]^, where undifferentiated cells represent the average Ct for the miRNA target minus average Ct for the reference miRNA in the cells cultured in GM.

### 2.6. Statistical Analysis

Data were reported as mean values ± error standard (SEM) for mRNA and miRNA expression levels. The distribution of continuous data was determined using the Shapiro–Wilk and Kolmogorov–Smirnov tests. Unpaired *t*-test with Welch’s correction or the two-tailed Mann–Whitney U test followed by Bonferroni multiple-comparison adjustment was used to detect significant differences for expression of target mRNA and miRNA between cells cultured in OM vs. cells cultured in GM, as appropriate. Data were analyzed using GraphPad Prism software version 9 (GraphPad, San Diego, CA, USA) for Windows. Values with *p*-values < 0.05 were considered statistically significant.

## 3. Results

### 3.1. Establishment of a TC Primary Cell Line and Putative TC1-SCs Line

**Figure 1:**
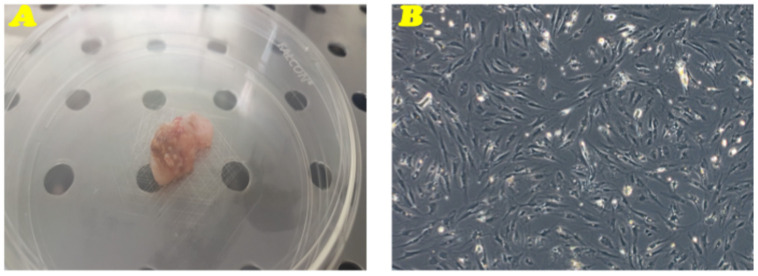
Biopsy sample and primary cell culture of TC. (**A**) Biopsy sample of TC, obtained by fine-needle aspiration. (**B**) Phase-contrast microscopy representative image of the TC1 primary cell culture. Original magnification 10×.

A primary culture of TC was isolated from subcutaneous adipose tissue of an ectopic calcification from a 16-year-old female patient with a new homozygous transition in intron 1 c.516-2a > g in the consensus region for splicing sites leading to skipping of exon 2 of the *GALNT3* gene, as previously described in a previous work [48]. The isolated primary culture was amplified until reaching the confluence in a 100 mm tissue culture dish and named as “TC1” (Figure 1).

**Figure 2:**
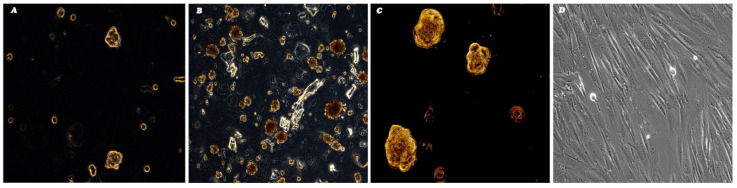
Formation of sphere derided from TC1 primary cell line and sphere adhesion after 48 h (**A**) and 14 days (**B**,**C**) under nonadherent, serum-free culture conditions. Monolayer expansion of spheres following reintroduction and reculturing in normal adherent conditions at 7 days from the isolation (**D**). The obtained putative SCs line was named as “TC1-SCs”. Observation in phase-contrast. Original magnification 10× (**B**,**D**) and 20× (**A**,**C**).

After cells were grown to reach the confluence, the monolayer was further subcultured to achieve the desired number of cells to cryopreserve the established primary culture and to isolate the putative SCs. Among the 2nd–4th passage, TC1 cells were detached and seeded in 6-well ultra-low attachment plates designed for the sphere-forming assay, considering the presence of a covalently bound hydrogel layer that successfully inhibits both cellular attachment to the substrate and the related anchorage-dependent cell signaling, thus mimicking the generation of a stressful condition that can only be sustained by SCs. Forty-eight hours after initiating this assay, cells appeared floating and separated spatially from one another (Figure 2A). After 14 days, large and bright spherical colonies called “spheres” were observed (Figure 2B,C). After this period, we isolated the large spherical colonies formed from each well of the plate and seeded immediately onto 60-mm diameter cell culture dishes under normal attachment conditions (Figure 2D). After 24 h, single cells within the colonies were found to attach to the plastic and their growth was monitored over time (Figure 2D). Therefore, we have obtained a putative SCs line, which has been named as “TC1-SCs”. This unique putative SC line was subcultured to obtain enough cells to cryopreserve this subpopulation and to perform analyses to characterize the stem-related features.

### 3.2. In Vitro Characterization of the Established Putative TC1-SCs Line

The characterization of the SC-like phenotype for the putative TC1-SCs line was carried out at the 5th passaging after isolation of the spheres.

**Figure 3:**
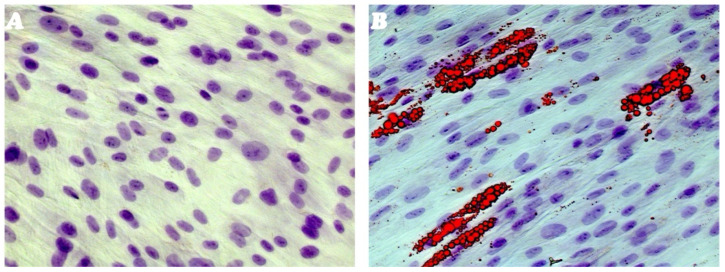
Adipogenic differentiation assay. Adipogenic differentiation of TC1-SCs line at 0 days (**A**) and after 14 days (**B**) of induction evaluated by cytochemistry with Oil Red O: in red, intracellular lipidic vesicles; in purple, nuclei counterstained by Toluidine Blue. Observation in Brightfield (LSM-900, ZEISS, Oberkochen, Germany). Original magnification 40×.

No adipogenic differentiation was observed in the undifferentiated TC1-SCs line, whereas AM-cultured cells showed adipogenesis after 14 days, revealing the accumulation of intracellular vesicles containing lipid droplets of variable size and shape (Figure 3A,B).

**Figure 4:**
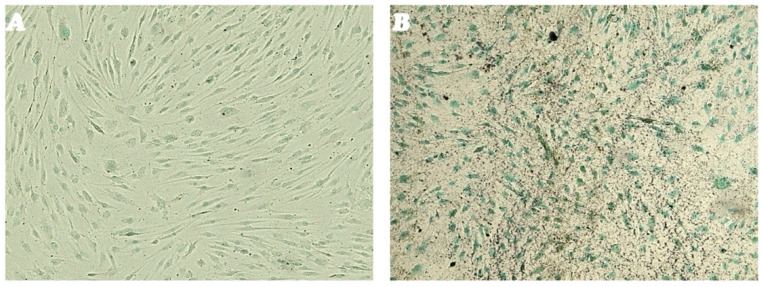
ALP and HA osteogenic differentiation. Osteogenic differentiation of the TC1-SCs line at day 14 (**A**) and at day 35 (**B**) of osteogenic induction evaluated by cytochemistry for ALP with Fast Red Violet B and for calcium mineralization with Von Kossa stain: in black, grainy deposits; and in green, nuclei counterstained with methyl green. Observation in Brightfield (LSM-900, ZEISS, Oberkochen, Germany). Original magnification 40×.

The isolated putative SCs line was then evaluated for its osteogenic differentiation capacity. Results obtained revealed that TC1-SCs exhibited a time-dependent increase in terms of the number and size of calcium mineral deposits (Figure 4B). In contrast, cells cultured in the control medium for the same time span did not show any mineralized nodules (Figure 4A). Interestingly, no ALP activity was detected neither in undifferentiated cells nor in cells differentiated toward the osteogenic lineage. These data were consistent with what was observed for the *ALP* gene expression during the osteogenic differentiation of TC1-SCs.

**Figure 5:**
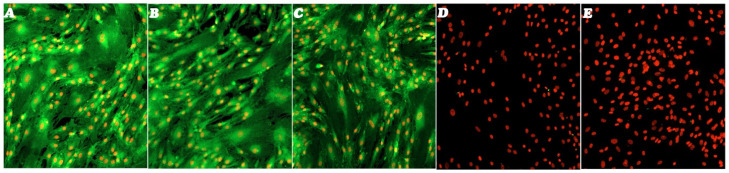
Immunofluorescence staining of the surface markers of MSCs and HSCs. Immunofluorescence staining of CD44 (**A**), CD90 (**B**) CD105 (**C**), CD34 (**D**), and CD45 (**E**) surface markers of the TC1-SCs line. Cells incubated with secondary antibodies only were used as immunofluorescence staining negative control for establishing background fluorescence and nonspecific staining of the primary antibodies. LSCM in traditional colors: green for MSC markers and bright red for nuclei. Original magnification: 10×.

Furthermore, a good rate of clonogenic efficiency of 21% for TC1-SCs was demonstrated in the CFU assay. Immunofluorescence staining revealed that the isolated TC1-SCs line was highly positive for MSC surface markers (CD44, CD90, and CD105) (Figure 5A,C), while not expressing hematopoietic SC (HSCs)–characterizing antigens (i.e., CD34 and CD45) (Figure 5D,E).

**Figure 6:**
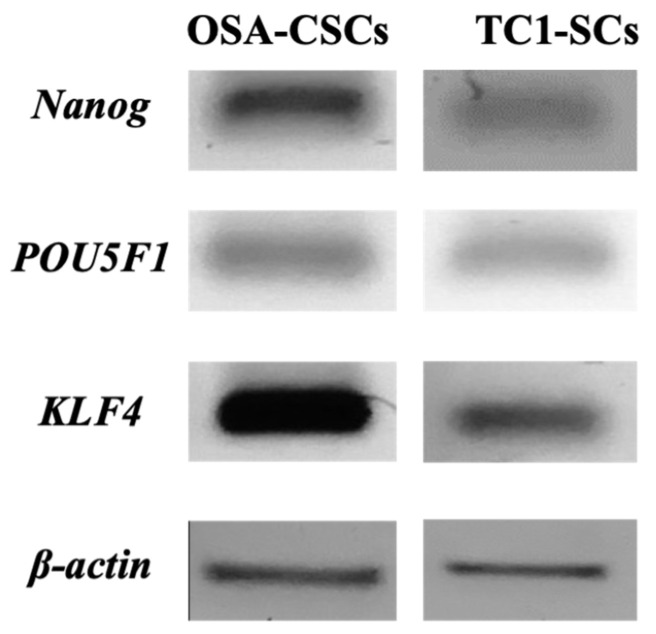
Expression levels of the ESC marker genes. In the isolated TC1-SCs line, gene expression evaluated by qualitative RT-PCR showed the expression of all the analyzed nuclear ESC-related marker genes (*POU5F1*, *Nanog*, and *KLF4*). The OSA-CSCs line was used as a positive control.

The expression of ESC-related marker genes (i.e., *POU5F1*, *Nanog*, and *KLF4*) in the isolated TC1-SCs line (Figure 6) was demonstrated by using qualitative RT-PCR analysis. Our observations were confirmed by positive results obtained with a previously established conventional OSA-CSCs line [47].

### 3.3. Differential Expression of Osteogenic Marker Genes and miRNAs in TC1, TC1-SCs, and PA24 During Osteogenic Induction

**Figure 7:**
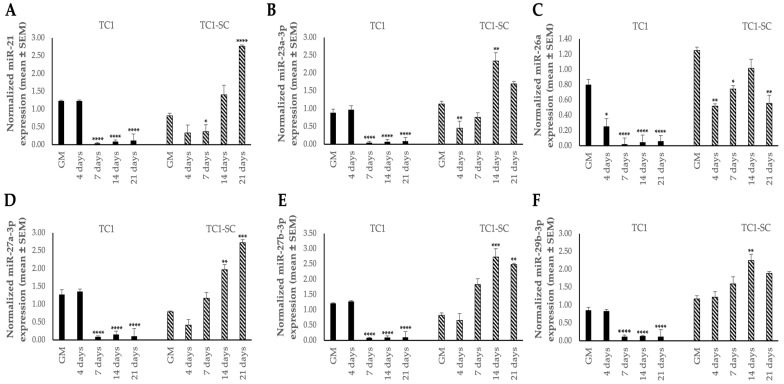
Expression levels of a panel of six miRNAs [miR-21 (**A**), miR-23a-3p (**B**), miR-26a (**C**), miR-27a-3p (**D**), miR-27b-3p (**E**), and miR-29b-3p (**F**)] evaluated during the osteogenic differentiation of the isolated TC1 and TC1-SCs cell lines by qPCR analyses. Values are expressed as mean ± SEM and normalized to small nucleolar RNA U6. * *p* < 0.05; ** *p* < 0.01; *** *p* < 0.001; **** *p* < 0.0001 as compared to cells cultured in GM for each cell line.

**Figure 8:**
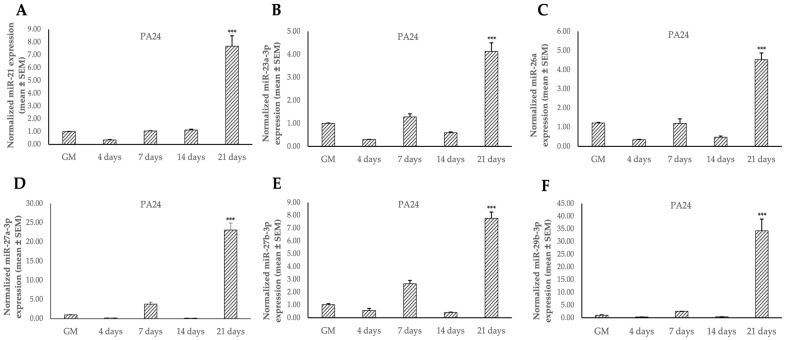
Expression levels of a panel of six miRNAs [(**A**), miR-23a-3p (**B**), miR-26a (**C**), miR-27a-3p (**D**), miR-27b-3p (**E**), and miR-29b-3p (**F**)] during the osteogenic differentiation of the PA24 cell line evaluated by qPCR analyses. Values are expressed as mean ± SEM and normalized to small nucleolar RNA U6. *** *p* < 0.001 as compared to as compared to cells cultured in GM.

Once the TC1-SCs cell line was established and characterized, we detected a preliminary miRNAs expression profile in undifferentiated and osteodifferentiated TC1, TC1-SCs, and PA24 by using qPCR. For this purpose, we selected a panel of six miRNAs (i.e., miR-21, miR-23a-3p, miR-26a, miR-27a-3p, miR-27b-3p, and miR-29b-3p) that were shown to participate in osteogenic differentiation [52], two of which were predicted in silico, using online software (i.e., TargetScan Release 8.0), to target the 3′UTR of the *GALNT3* mRNA (i.e., miR-27a-3p and miR-27b-3p). qPCR showed an miRNA signature that was specific to the isolated TC1-SCs line compared to the primary TC cell line (Figure 7). Collectively, five out of six selected miRNAs (i.e., miR-21, miR-23a-3p, miR-27a-3p, miR-27b-3p, and miR-29b-3p) exhibited inverse expression patterns during osteogenic differentiation between TC1 and TC1-SCs (Figure 7). Accordingly, we found increased expression levels for all these miRNAs in the PA24 cell line. However, we detected an inverse expression pattern of miR-26a during osteogenic differentiation between both the TC cell lines and PA24, identifying a significant downregulation for the miR-26a expression levels during the osteogenic differentiation of either the primary TC1 and TC1-SCs lines (Figure 7C and Figure 8C).

In summary, we identified an miRNA expression profile that is characteristic of the established TC1-SCs in vitro model.

**Figure 9:**
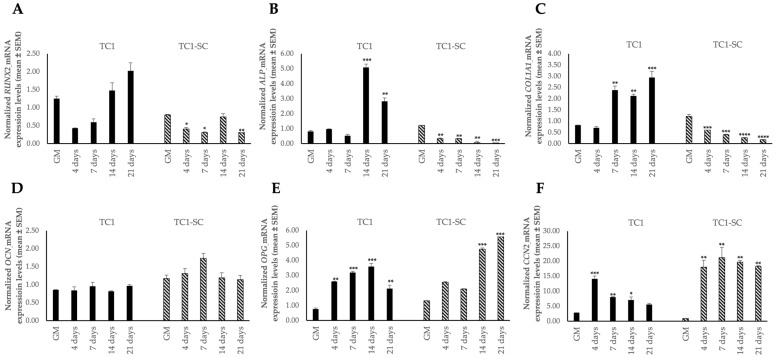
Expression levels of osteogenic marker genes *RUNX2* (**A**), *ALP* (**B**), *COL1A1* (**C**), *OCN* (**D**), *OPG* (**E**), and *CCN2* (**F**) during the osteogenic differentiation in the isolated TC1 and TC1-SCs cell lines. Values are expressed as mean ± SEM and normalized to the housekeeping gene *GAPDH*. * *p* < 0.05; ** *p* < 0.01; *** *p* < 0.001; **** *p* < 0.0001 as compared to cells cultured in GM for each cell line.

**Figure 10:**
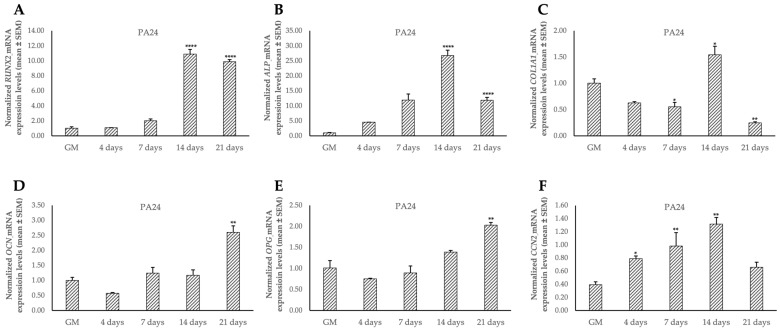
Expression levels of osteogenic marker genes *RUNX2* (**A**), *ALP* (**B**), *COL1A1* (**C**), *OCN* (**D**), *OPG* (**E**), and *CCN2* (**F**) during the osteogenic differentiation in PA24 cell line. Values are expressed as mean ± SEM and normalized to the housekeeping gene *GAPDH*. * *p* < 0.05; ** *p* < 0.01; **** *p* < 0.0001 as compared to cells cultured in GM.

Moreover, we also investigated the expression of osteogenic marker genes (i.e., *RUNX2*, *ALP*, *COL1A1*, *OCN*, *OPG*, and *CCN2*) in the TC1, TC1-SCs, and PA24 cell lines. Data obtained from the qPCR analysis reported for the first time a characteristic expression signature of the osteogenic genes in the TC1-SCs compared both with the primary cell line obtained and with the PA24 cell line (Figure 9 and Figure 10).

## 4. Discussion

TC is a rare hereditary disease of phosphate metabolism characterized by the formation of heterogenous multilobulated ectopic calcified lesions mainly located in the periarticular soft tissue spaces [8]. This metabolic dysfunction is not completely understood so far, and the differential diagnosis with similar-appearing lesions (i.e., chronic kidney disease (CKD), myositis ossificans, tophaceous gout, dystrophic) is difficult when only imaging diagnostic procedure alone is made [8].

Due to the rarity of this disabling condition, there is very limited data available on the best therapeutic approach to treat ectopic soft tissue calcification in TC, which includes phosphate-lowering therapies (i.e., low phosphate diet, medications inhibiting dietary phosphate intestinal absorption, and acetazolamide), anti-inflammatory therapies (i.e., steroidal anti-inflammatory drugs and corticosteroids), anti-mineralization therapies (i.e., sodium thiosulfate), the use of bisphosphonates, calcium-channel blockers, laser therapy, and surgical resection. To date, this latter combined with the use of aluminum hydroxide (a phosphate binder), phosphate-restricted diets, and acetazolamide (a carbonic anhydrase inhibitor) has so far been the most effective therapy for TC [53,54]. However, surgical resection outcomes are highly variable, and this approach is pretty reserved for those TC patients who have significant impairment of daily activities or chronic drainage and inflammation [1]. Furthermore, since *FGF23* or *GALNT3* mutations result in decreased intact FGF23 serum levels, the ideal treatment for these patients could be hormone replacement therapy with FGF23. However, many challenges need to be fully addressed before FGF23 replacement therapy can become a routine treatment for TC [8].

Over the past decade, several models have been generated to replicate the biochemical abnormalities of TC even though resembling the human TC phenotype remains challenging so far. In the mouse model developed by Ichikawa and colleagues [55], the ablation of *GALNT3* in exons 2 and 3 led to decreased circulating intact Fgf23 levels with hyperphosphatemia, despite that high levels of Fgf23 were found in bone [55]. This mouse model was further characterized by abnormally 1α,25(OH)_2_D_3_ levels, reduced ALP activity, and no ectopic calcifications. In another TC mouse model, a Trp589Arg *GALNT3* mutation was generated by N-ethyl-N-nitrosourea (ENU) mutagenesis, resulting in hyperphosphatemia associated with decreased intact FGF23 levels, increased 1α,25(OH)_2_D_3_ levels, and periarticular calcified masses [56].

Despite that murine models are being extensively used for studying human bone-related diseases, skeletal regulatory mechanisms are diverged at some stages between humans and mice [57]. More than half of cellular signaling pathways have been conserved over the evolution between these two species, such as Bone Morphogenetic Protein (BMP), Hedgehog, FGF, and Notch and transcriptional regulators of osteogenesis, such as SOX9 and RUNX2 [57]. However, mice and humans have been demonstrated to diverge in different members of the Wnt family, including CXXC Finger Protein 4 (CXXC4), (sclerostin) SOST, and deoxyribonucleic acid (DNA)JB6 [57].

In addition to murine models, the use of in vitro primary cell models obtained directly from patients might be helpful in eviscerating the biological mechanisms underlying the pathology. Regarding in vitro model systems that are used for studying TC, in mouse osteoblastic MC3T3.E1 cells, FGF23/Klotho has been demonstrated to modulate the expression of genes that are involved in the osteoblastic differentiation and mineralization process, probably via FGF receptor 1 (FGFR1) signaling [58].

A previous study by Masi et al. [48] investigated how the GALNT3/FGF23/Klotho axis might regulate the functions of osteoblasts using an in vitro preosteoblastic culture isolated from a subcutaneous adipose tissue of a TC patient affected by a previously undescribed inactivating mutation in the *GALNT3* gene. The study found that the cell line obtained from the patient exhibited a higher ability to form HA crystals earlier and more abundantly than cells obtained from healthy individuals, suggesting that the *GALNT3* gene mutation observed in this patient might led to reduced glycosylated activity by GALNT3 with a consequent increase in FGF23 proteolysis, resulting in large amounts of inactive C-terminal FGF23 fragments in sera and low amounts of the intact molecule.

Over the last few years, emerging evidence highlighted the presence not only of the SCs population potentially involved in pathological mechanisms contributing to bone disorders, but also of a CSC subpopulation within the tumor bulk, including bone-related cancer (i.e., osteosarcoma), that could be responsible for tumor initiation, formation, metastasis, and poor prognosis of cancer patients [59,60,61,62,63,64]. Therefore, it has been postulated that their investigation could lead to identifying novel targets for the development of innovative anticancer approaches.

In the present study, we have shown that TC can also contain an SC subpopulation with the ability to anchorage-independently grow and to survive under stress conditions, which could aid in eviscerating the mechanisms underlying TC pathogenesis and recurrence.

The expression of osteogenic markers genes (*RUNX2*, *ALP*, and *COLIA1*) markedly reduced during the osteogenic differentiation process in the TC1-SCs line compared both to the primary TC line and the WT counterpart (PA24 cell line), suggesting that other unexplored pathways or mechanisms may be responsible for the development of the calcifications associated with TC. The increased expression of *OPG* mRNA levels, the decoy receptor that blocks nuclear factor κB ligand (RANKL) thus preventing RANK activation and osteoclastogenesis [65], and the absence of *RANKL* expression, in both the TC-derived cell lines compared with the primary wild-type subcutaneous SC line, could at least in part clarify the pathogenesis of development of prominent periarticular calcified masses in TC patients. However, no study has focused its attention on the role of the RANKL/RANK/OPG axis in TC so far.

Different investigations have been carried out to explore the role of miRNAs in the processes of skeletal development and their involvement in the evolution of bone diseases [66,67,68,69]. However, as far as we know, no previous studies have evaluated miRNAs in TC. Therefore, this study represents the first evidence of an miRNA expression pattern characteristic for the isolated TC1-SCs. In particular, miR-26a resulted to be downregulated both in primary TC1 and TC1-SC cell lines, with respect to the non-TC control cell line. In relation to this finding, miR-26a was shown as an important miRNA with the ability to regulate osteogenic differentiation of adipose tissue–derived mesenchymal SCs (ADSCs). In a study by Li et al. [70], miR-26a overexpression inhibited osteogenic differentiation, while its downregulation induced osteogenesis in mouse ADSCs. Furthermore, the authors revealed that the increased miR-26a expression hindered mouse ADSCs osteogenic differentiation by directly targeting the *Wnt5a* mRNA, thus suppressing the Wnt/Ca^2+^ signaling pathway. Further former research has shown that miR-26a can affect osteogenesis via the regulation of Wnt, through the inhibition of GSK-3β, and BMP/SMAD1 signaling pathways [71,72]. An additional study by Kim and colleagues [73] examined the relevance of miR-26a also in osteoclastogenesis, identifying that its expression levels were enhanced during the terminal stage of RANKL-induced osteoclastogenesis. Functional analysis suggested that the ectopic expression of miR-26a negatively regulated the osteoclast differentiation of bone marrow–derived macrophage-like cells by reducing the expression of *CCN2* mRNA. The latter was not only demonstrated to be a master regulator of osteoclastogenesis through the dendritic cell–specific transmembrane protein (DC-STAMP), but its importance was also demonstrated in endochondral ossification in which it stimulates the proliferation and maturation of osteoblasts and chondrocytes [74]. Our finding demonstrated that *CCN2* mRNA expression was significantly increased during the osteogenic differentiation in both TC-derived in vitro models as compared to the control cell line PA24. Since it has been already displayed the negative feedback regulation of miR-26a on CCN2 and the critical role of CCN2 in regulating osteoblastogenesis, it can be assumed that the enhancement of miR-26a expression levels could be the basis for the design of novel approaches to prevent or to contrast the formation of multiple large amorphous calcium phosphate deposits around large joints typically observed in TC.

**Figure 11:**
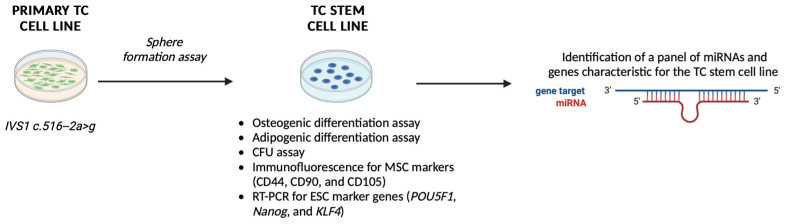
Schematic representation of the results obtained in this study. Abbreviation: TC: tumoral calcinosis; CFU: colony forming unit; MSC: mesenchymal stem cell; ESC: embryonic stem cell. Figure created using BioRender.com, accessed on 15 February 2025.

In conclusion, we have, for the first time, established and characterized an SC line from a TC patient harboring a novel *GALNT3* mutation, which could potentially represent a valid in vitro model for studying the causes of ectopic calcification associated with TC (Figure 11). In addition, a preliminary signature of miRNAs specific to the TC1-SCs line was identified for the first time in our study. Additional investigations, aimed to identify further miRNAs being differentially expressed between the primary TC line and non-TC control line, are currently ongoing. Finally, we are investigating whether the downregulation of miR-26a could be responsible for the periarticular calcium deposits associated with this inherited disease through the regulation of CCN2 expression.

Taken together, the existence of an SC subpopulation inside TC lesions, as well as the study of the biological mechanisms underlying the TC pathogenesis, may be important steps for the comprehension of TC pathogenesis, thereby paving the way for the identification of new diagnostic and therapeutic targets for TC in the future.

## Figures and Tables

**Table 1:** List of primers and TaqMan probes used in RT-PCR and qPCR analyses.

Gene	Oligonucleotides	Primer Sequence (5′–3′)	Amplicon Size (bp)	Ta (°C)
*β* *-actin*	Forward Reverse	AGCCTCGCCTTTGCCGA CTGGTGCCTGGGGCG	174	54
*POU5F1*	Forward Reverse	GGGAGAGCTAGGGAAAGA TCCTTCCTTAGTGAATGAAGAACT	77	60
*Nanog*	Forward Reverse	CCCAGCTGTGTGTACTCAAT GGTTCAGGATGTTGGAGAGTT	87	60
*KLF4*	Forward Reverse	CGGGAAGGGAGAAGACACT AGTCGCTTCATGTGGGAGA	79	60
*GAPDH*	Forward Probe Reverse	AATCCGTTGACTCCGACCTTC /56-FAM/CCACATCGC/ZEN/TCAGACACCATGGG/3IABkFQ/ ACAGTACAGCCGCATCTTC	179	58
*ALP*	Forward Probe Reverse	CATACAGGATGGCAGTGAAGG /56-FAM/TTCTTGTCT/ZEN/GTGTCACTCAGCATGGG/3IABkFQ/ CCCGTGGCAACTCTATCTTTG	78	62
*RUNX2*	Forward Probe Reverse	CTCACGTCGCTCATTTTGC /56-FAM/TCTTTTGGA/ZEN/TCCGAGCACCAGCC/3IABkFQ/ AGGGACTATGGCATCAAACAG	135	58
*OPG*	Forward Probe Reverse	GAAGGTGAGGTTAGCATGTCC /56-FAM/TGTGAAAAC/ZEN/AGCGTGCAGCGG/3IABkFQ/ GCAACACAGCTCACAAGAAC	151	60
*COL1A1*	Forward Probe Reverse	CCAGCCACAAAGAGTCTACAT ATGTCCCACCAATCACCTGCGTA CGGGTTTCCACACGTCTC	202	58
*CCN2*	Forward Probe Reverse	GAGCAGCTGCAAGTACCA /56-FAM/TGTGCAGCA/ZEN/TGGACGTTCGTCT/3IABkFQ/ ACTCCTCGCAGCATTTCC	140	54
*OCN*	Forward Probe Reverse	AGCTCACACACCTCCCT /56-FAM/TCCCCTACC/ZEN/CGGATCCCCT/3IABkFQ/ 5′-CGCTACCTGTATCAATGGCTG-3′	77	57

Ta: annealing temperature; bp: base pair.

## Data Availability

The data presented in this study are available on request from the corresponding author.
